# Outer membrane proteins analysis of *Shigella sonnei* and evaluation of their antigenicity in *Shigella* infected individuals

**DOI:** 10.1371/journal.pone.0182878

**Published:** 2017-08-28

**Authors:** Hemavathy Harikrishnan, Kirnpal Kaur Banga Singh, Asma Ismail

**Affiliations:** 1 Department of Medical Microbiology & Parasitology, School of Medical Sciences, Health Campus, Universiti Sains Malaysia, Kelantan, Malaysia; 2 Office of the Vice-Chancellor, Universiti Sains Malaysia, Penang, Malaysia; 3 Institute for Research in Molecular Medicine (INFORMM), Universiti Sains Malaysia, Kelantan, Malaysia; Bharathidasan University, INDIA

## Abstract

Bacillary dysentery caused by infection with *Shigella* spp. remains as serious and common health problem throughout the world. It is a highly multi drug resistant organism and rarely identified from the patient at the early stage of infection. *S*. *sonnei* is the most frequently isolated species causing shigellosis in industrialized countries. The antigenicity of outer membrane protein of this pathogen expressed during human infection has not been identified to date. We have studied the antigenic outer membrane proteins expressed by *S*. *sonnei*, with the aim of identifying presence of specific IgA and IgG in human serum against the candidate protein biomarkers. Three antigenic OMPs sized 33.3, 43.8 and 100.3 kDa were uniquely recognized by IgA and IgG from patients with *S*. *sonnei* infection, and did not cross-react with sera from patients with other types of infection. The antigenic proteome data generated in this study are a first for OMPs of *S*. *sonnei*, and they provide important insights of human immune responses. Furthermore, numerous prime candidate proteins were identified which will aid the development of new diagnostic tools for the detection of *S*. *sonnei*.

## Introduction

*Shigella sonnei*, the causative agent of shigellosis (also known as bacillary dysentery), is a Gram-negative human facultative pathogen where the infection acquired by faecal-oral route and enters the human body via the ingestion of contaminated food and water [1]. The bacteria are highly infectious, since as few as 10 to 100 microorganisms are capable to cause disease as they are able to survive in acidic environment in the stomach [2, 3]. Shigellosis has been reported as a disease of all age groups but it is commonly seen in pediatric patients [4–6]. It causes approximately 1.1 million people die each year, and 60% of diarrhea-associated mortality among children’s below five years old in developing countries [7, 8]. The pathogenesis of shigellosis includes inflammation, ulceration, haemorrhage, tissue destruction and fibrosis of the colonic mucosal. These clinical symptoms will lead to abdominal pain and diarrhea [9].

Although antibiotics are used for treatment in all cases of shigellosis but due to the global emergence of drug resistance, the choice of antimicrobial agents for treating shigellosis is limited[9]. The *Shigella* resistance locus (SRL) has been identified in *Shigella* strains which mediates resistance to antibiotics[10].

Despite all these, the presence of this organism is still detected using traditional diagnostic methods such as culture and biochemical test. This traditional method, which is time consuming and may take at least 48 to 72 hours or even longer to obtain a result. The early diagnosis and treatment are important to control the outbreak of this contagious disease. [11]. Therefore it is crucial to develop a new, fast, specific and sensitive, and economical test for rapid detection of *S*. *sonnei* infections. Development of such a test would require the identification of specific antigenic proteins and OMPs that are recognized by host antibodies [12]. OMPs of *Shigella* are suitable antigenic proteins due to their well-known role in the molecular pathogenesis of shigellosis [13]^.^ Besides that, the OMPs due to their location, have been known to elicit a host immune response and are also categorized as virulence factors [14]. Some proteins in OMPs of the bacterium are exposed on the cell surface and may influence the physiological functions of the tissue, contributing to the mechanisms of pathogenicity and development of inflammatory response [15, 16].

This study was conducted to determine the presence of antigenic and specific OMPs in *S*. *sonnei* that are recognized by host antibodies and to identify the presence of specific Ig in patients’ sera against the candidate protein(s). This protein(s) can be utilized as a potential biomarker test against *S*. *sonnei*.

## Materials and methods

### Collection and culture of bacterial strains

The clinical strain of *S*. *sonnei* SH080, SH039 and SH040 used in this study were obtained from the Department of Medical Microbiology & Parasitology, School of Medical Sciences, Universiti Sains Malaysia, Malaysia. These clinical isolates were obtained from patients that were culture positive for *S*. *sonnei*. A reference strain, *S*. *sonnei* ATCC 25931, was used in this study and was the standard organism for protein profiling in this study. *S*. *sonnei* ATCC 25931 and the clinical isolates were maintained in trypticase soy broth with 20% glycerol and kept at -20°C [17].

### Ethics statement

Samples were collected from patients of either sex admitted in the ward or patients attending the outpatient clinics at the Hospital Universiti Sains Malaysia, Kelantan, within 3 weeks of the cultural diagnosis of pathogens. The subjects ranged from 12 to 50 years of age and had feelings of illness due to diarrhea. Patients with mixed bacterial infection were excluded. The bacterial strains were identified at the species level using a commercial biochemical differentiation kit (API CAMPY, bioMe´rieux). The use of human sample was approved by the Human Ethical Committee of Universiti Sains Malaysia (USMKK/PPP/JEPeM/248.3(10)). Written informed consent was obtained from all subjects before participation in the study.

### Outer membrane proteins preparation

Bacteria were grown at 37°C in nutrient broth for 18 hours and harvested by centrifugation. OMPs were extracted using a previously described method [12, 18]. Briefly, bacteria were grown in 2 L of nutrient broth and incubated in a shaker (Forma Orbital Shaker, Model-420, USA) at 37°C at 200 rpm for 18 h. Cells were harvested by centrifugation at 15,900 x g for 30 minutes and resuspended in 8 ml of 0.01M HEPES (N-2 hydroxyethylpiperazine-N’-2ethanesulfonicacid) buffer (pH7.4) containing 8 μl of 10 mM DNAse (Sigma, USA), 8 μl of 10 mM RNAse (Sigma, USA) and 800 μl of 100 mM phenylmethylsulfonyl fluoride (~0.2 mm in diameter, BDH Chemical Ltd., UK). Bacterial cells were disrupted by vortexing with glass beads (~0.2 mm in diameter, BDH Chemical Ltd.) for 1.5 hours with 1 minute alternate on ice until 95% lysis was achieved. The cell lysate was aspirated and the glass beads washed with 0.01 M HEPES buffer until the washings were cleared. The disrupted cells were centrifuged at 7800 x g, 4°C for 15 minutes to remove the undisrupted cells. Then, the supernatant was centrifuged with an ultracentrifuge (Hitachi, Model CP 80MX) at 145,100 x g, 4°C for 1 hour (using rotor type P40 ST) to separate the envelope fractions. The cell envelope sediment was extracted with 0.01 M HEPES containing 4% Triton X-100 (Bio-Rad, USA) to detach the cytoplasmic inner membrane from outer membrane. The mixture was incubated at room temperature for 10 min. The insoluble OMPs was pelleted using ultracentrifuge (Hitachi, Model CP 80MX) at 181,800 x g, 4°C for 1 hour (using rotor type P5S ST2). The pellet was resuspended with 4 ml of 30 mM Tris HCl, pH 8.0. The total protein concentrations in the samples were determined using a Bradford assay method (Bio-Rad, USA).

### Profiling of the OMPs of *S*. *sonnei* using sodium dodecyl sulphate polyacrylamide gel electrophoresis

In this study, discontinuous SDS-PAGE protocol was used to analyse protein profiles of *S*. *sonnei* based on differences in their molecular weight. Protein samples containing 30 ug of proteins were resolved on10% polyacrylamide gel by applying 35 mA current for 3 hr using PROTEAN II xi (Bio-Rad, USA). The OMPs obtained was re-suspended with sample buffer containing 0.1% SDS and β-mercaptoethanol (10%). The OMPs profile of *S*. *sonnei* was observed via SDS-PAGE gel stained with Coomassie brilliant blue (Bio-Rad, USA).

### Western blot

Western blotting was performed using standard protocol [12, 18]. OMPs were resolved via SDS-PAGE and electroblotted from the gel to a 0.45 μm pore size nitrocellulose membrane by electrophoretic transfer using Bio-Rad Transblot apparatus (Bio-Rad Laboratories, USA). The electroblotting condition was set at 100 V for 4 hours according to the manufacturer’s instruction. The nitrocellulose membrane was then blocked with 3% skim milk for 30 min at room temperature to block the non-specific protein binding sites. The blocked nitrocellulose membranes were cut into strips and incubated overnight with 1:100 dilutions of human serum as the primary antibody. Sera used in this study as follows: from patients positive for *S*. *sonnei* infection; and patients from other enteric infections due to *Salmonella* spp., *Aeromonas hydrophila*, EPEC, *Salmonella* Typhi and *Campylobacter jejuni*. After primary antibody incubation, the strips were washed with PBS-T (0.05% Tween 20) 6 times for 10 minute each. It was then incubated with rabbit anti-human IgA and IgG (Sigma, USA) conjugated with alkaline phosphatase for 2 hours at room temperature with constant shaking. The strips were washed with PBS-T as above and then developed with AP-conjugated substrate for 30 minute. The enzyme reaction was stopped by rinsing the strips with distilled water.

#### Determining the presence of antigenic and specific protein(s) of *S*. *sonnei* in OMPs using SDS-PAGE and Western blotting

The protein bands were determined by using image analyzer (SYNGENE Bio Imaging System, Japan) to measure the molecular weight of the bands against the reference standard. The analysis involved construction of a standard calibration curve according to the value of the standard molecular weight markers. Subsequently, the bands were located from the captured image of the SDS-PAGE profile and their corresponding molecular weight was determined. Comparison against positive control sera and elimination of the cross-reactive proteins with sera from patients with other enteric infections were performed to determine the specific and antigenic protein(s) expressed by *S*. *sonnei*.

The main aim of this study was to determine the presence of OMPs of *S*. *sonnei* that are both antigenic and specifically recognized by serum from humans infected with *S*. *sonnei*. The following 9 different types of serum were used for Western blot analysis: 4 (SS01, SS02, SS03 and SS04) from culture-positive cases of *S*. *sonnei*; 5 from patients infected with *Salmonella* spp. (SASP01), *Aeromonas hydrophila* (AH01), EPEC (E01), *Salmonella* Typhi (ST01) and *Campylobacter jejuni* (CAMPY01) respectively. Before checking the antigenicity and specificity of the OMPs of *S*. *sonnei* by Western blot, the total immunoglobulin profiles of all the sera as well as the specific immunoglobulin profiles against *S*. *sonnei* OMPs were tested. Total immunoglobulin was tested via dot enzyme immunoassay (EIA) by dotting the sera and probed with IgA and G as primary antibody and the complex was visualized using anti-human immunoglobulin A and G conjugated with alkaline phosphatase. The specific immunoglobulin profiles against the antigenic protein were determined by dotting OMPs of *S*. *sonnei* and probed individually with IgA and IgG. Since all the tested sera gave high positive reaction with OMPs of the clinical isolate, all the sera were selected for Western blot analysis in order to determine the antigenicity of each protein present in the OMPs mixture.

## Results

### Determination of presence of antibodies (IgA and IgG) against SH080, *Shigella sonnei* OMPs

**Fig 1:**
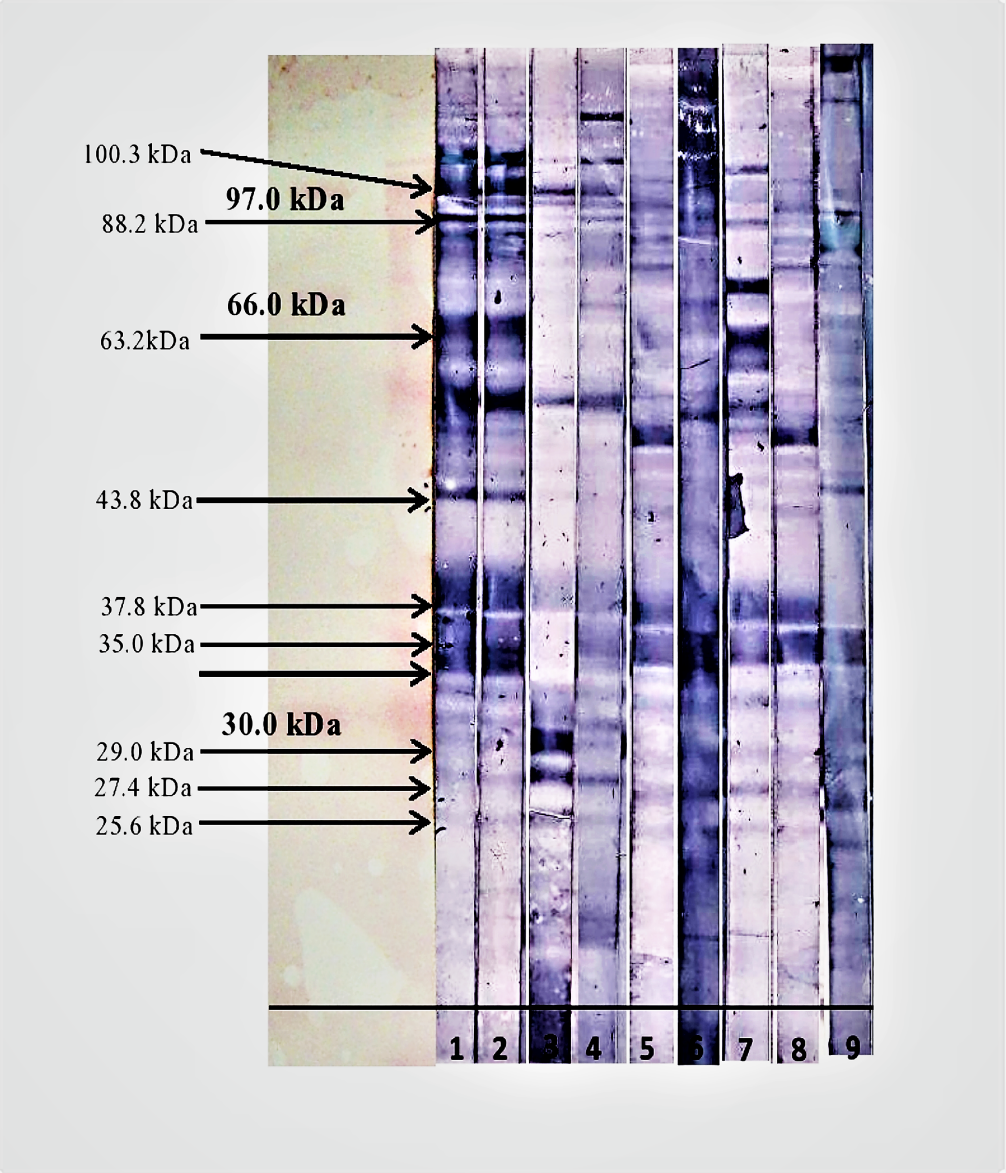
Western blot result of OMPs of *S*. *sonnei* probed with anti-human IgA. Strip-1: *S*. *sonnei* (SS001), Strip-2: *S*. *sonnei* (SS002), Strip-3: *S*. *sonnei* (SS003), Strip-4: *S*. *sonnei* (SS004), Strip-5: *Salmonella* species (SASP01), Strip-6: EPEC (E01), Strip-7: *Salmonella* Typhi (ST01), Strip-8: *Aeromonas hydrophila* (AH01), Strip-9: *Campylobacter jejuni* (CAMPY01).

**Fig 2:**
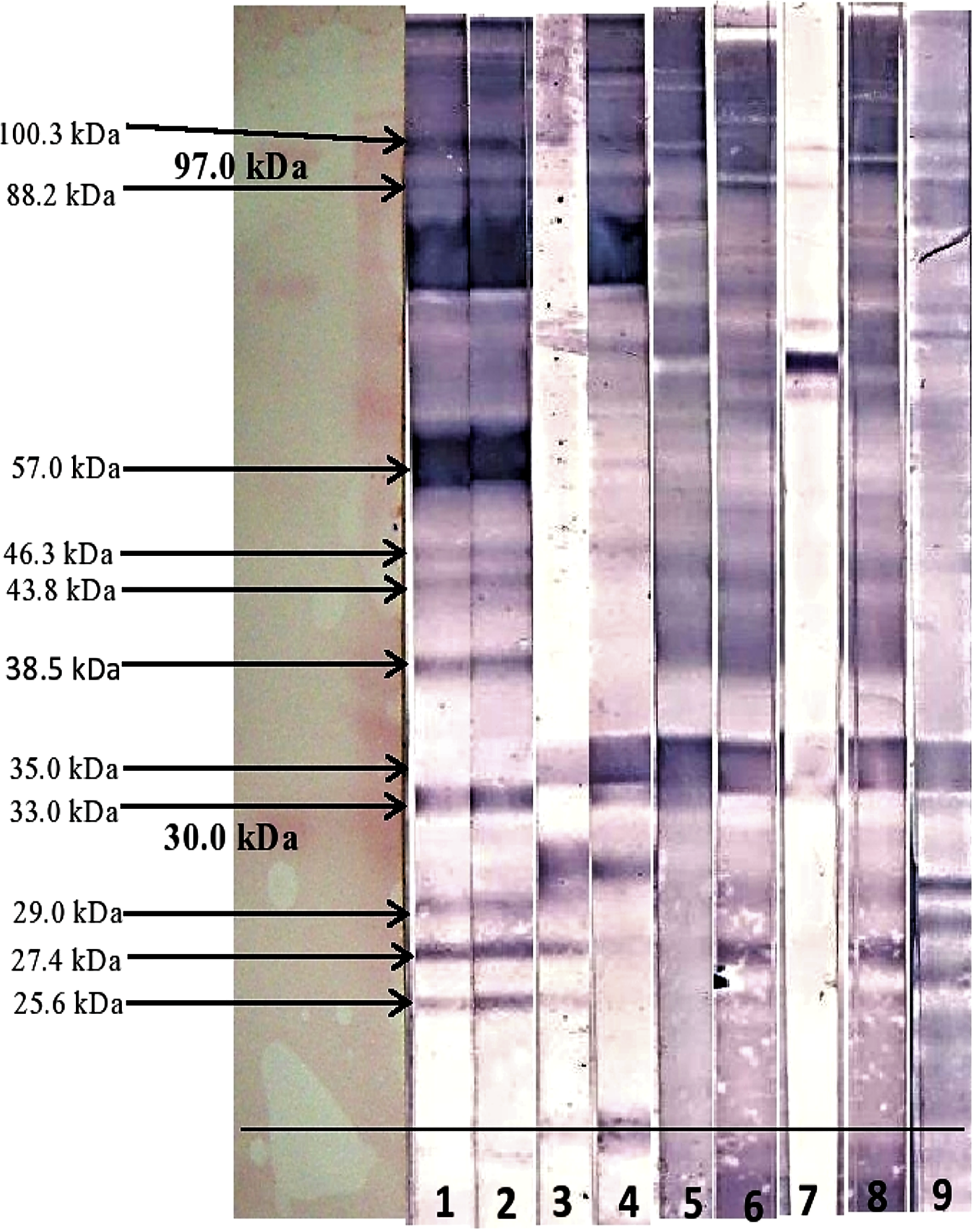
Western blot result of OMPs of *S*. *sonnei* probed with anti-human IgG. Strip-1: *S*. *sonnei* (SS001), Strip-2: *S*. *sonnei* (SS002), Strip-3: *S*. *sonnei* (SS003), Strip-4: *S*. *sonnei* (SS004), Strip-5: *Salmonella* species (SASP01), Strip-6: EPEC (E01), Strip-7: *Salmonella* Typhi (ST01), Strip-8: *Aeromonas hydrophila* (AH01), Strip-9: *Campylobacter jejuni* (CAMPY01).

Figs 1 and 2 show the immunoblot profile of all the four sera from patients infected with *S sonnei* against the IgA and IgG isotype respectively. A total of 13 antigenic bands were observed in the OMPs of *S*. *sonnei* when probed with shigellosis patients’ sera as shown in Table 1. Of these 13 antigenic bands, 11 proteins (molecular weight 25.6, 27.4, 29.0, 33.3, 35.0, 37.8, 43.8, 57.0, 63.2, 88.2 and 100.3 kDa) were recognized against the anti-human IgA when probed with sera SS001 and SS002. However, with SS003 serum, only seven antigenic bands (25.6, 27.4, 29.0, 37.8, 43.8, 57.0 and 100.3 kDa) were recognized against anti-human IgA, whereas, with SH004 only five antigenic bands (27.4, 29.0, 57.0, 88.2 and 100.3 kDa) were recognized. When tested with anti-human IgG, there were ten antigenic bands (25.6, 27.4, 29.0, 33.3, 38.5, 43.8, 46.3, 57.0, 88.2 and 100.3 kDa) with sera SS001 and SS002 whereas, sera SS003 and SS004 only recognized four (25.6, 27.4, 29.0 and 35.0 kDa) and six (27.4 kDa, 33.3 kDa, 35.0 kDa, 46.3 kDa, 88.2 kDa and 100.3 kDa) antigenic proteins respectively.

**Table 1:** Summary of cross-reacting immunogenic OMPs of *S*. *sonnei* when probed with anti-human IgA.

Molecular weight of the bands	Immunogenic bands when probed with anti-human IgA				Immunogenic bands that did/did not cross-reacted with non-shigellosis sera				
	SS001	SS002	SS003	SS004	SASP01	E01	ST01	AH01	CAMPY01
25.6 kDa	+	+	+	ND	CR	CR	CR	CR	ND
27.4 kDa	+	+	+	+	CR	CR	CR	CR	ND
29.0 kDa	+	+	+	+	Do not cross react				
33.3 kDa	+	+	ND	ND	Do not cross react				
35.0 kDa	+	+	ND	ND	CR	CR	CR	CR	CR
37.8 kDa	+	+	+	+	CR	CR	CR	CR	ND
43.8 kDa	+	+	+	ND	Do not cross react				
57.0 kDa	+	+	+	+	ND	CR	CR	ND	ND
63.2 kDa	+	+	ND	ND	ND	ND	CR	ND	ND
88.2 kDa	+	+	ND	ND	Do not cross react				
100.3 kDa	+	+	+	+	Do not cross react				

+ Indicate the observation of antigenic band. ND: Not detected; CR: Cross-reacted with non-shigellosis sera.

The study demonstrated that, some protein bands were exclusively recognized by only one subtype of antibody by all the *S*. *sonnei* sera. For example, two proteins were exclusively detected when probed with anti-human IgA (37.8 and 63.2 kDa) and another two proteins (46.3 and 38.5 kDa) were exclusively detected with anti-human IgG. The study also revealed the recognition of both isotype of antibodies (IgA and IgG) against eight protein bands of *S*. *sonnei* (25.6, 27.4, 29.0, 33.3, 43.8, 57.0, 88.2 and 100.3 kDa) when tested with sera SS001 and SS002.

Majority of the other antigenic bands were cross-reacting with most of the non-shigellosis sera tested. When evaluating for cross reactivity, among the 11 antigenic proteins seen in the shigellosis sera against IgA subtype only five bands (29.0, 33.3, 43.8, 88.2 and 100.3 kDa) did not cross-reacted with non-shigellosis sera as shown in Table 1. Another five bands (33.3, 38.5, 43.8, 57.0 and 100.3 kDa) were seen in the shigellosis sera against IgG subtype did not cross reacted with non-shigellosis sera as shown in Table 2. However, four protein bands (25.6, 29.0, 46.3 and 88.2 kDa) were only cross-reacting with only one non-shigellosis serum. The band 25.6 kDa cross-reacted with sera *Aeromonas hydrophila* while the band 29.0 kDa cross-reacted with sera *Campylobacter jejuni*. However, protein of 63.2 kDa in size was only cross-reacting with one non-shigellosis serum (ST01, *Salmonella* Typhi).

**Table 2:** Summary of cross-reacting immunogenic OMPs of *S*. *sonnei* when probed with anti-human IgG.

Molecular weight of the bands	Immunogenic bands when probed with anti-human IgG				Immunogenic bands that did/did not cross-reacted with non-shigellosis sera						
	SS001	SS002	SS003	SS004	SASP01	E01		ST01	AH01		CAMPY01
25.6 kDa	+	+	+	ND	ND	ND		ND	CR		ND
27.4 kDa	+	+	+	+	CR	ND		ND	CR		CR
29.0 kDa	+	+	+	ND	ND	ND	ND		ND		CR
33.3 kDa	+	+	ND	+	Do not cross react						
35.0 kDa	ND	ND	+	+	CR	CR		ND	CR		CR
37.8 kDa	+	+	ND	ND	Do not cross react						
43.8 kDa	+	+	ND	ND	Do not cross react						
57.0 kDa	+	+	ND	+	CR	ND		ND	ND		ND
63.2 kDa	+	+	ND	ND	Do not cross react						
88.2 kDa	+	+	ND	+	CR	ND		ND	ND	ND	
100.3 kDa	+	+	ND	+	Do not cross react						

+ Indicate the observation of antigenic band. ND: Not detected; CR: Cross-reacted with non-shigellosis sera.

**Fig 3:**
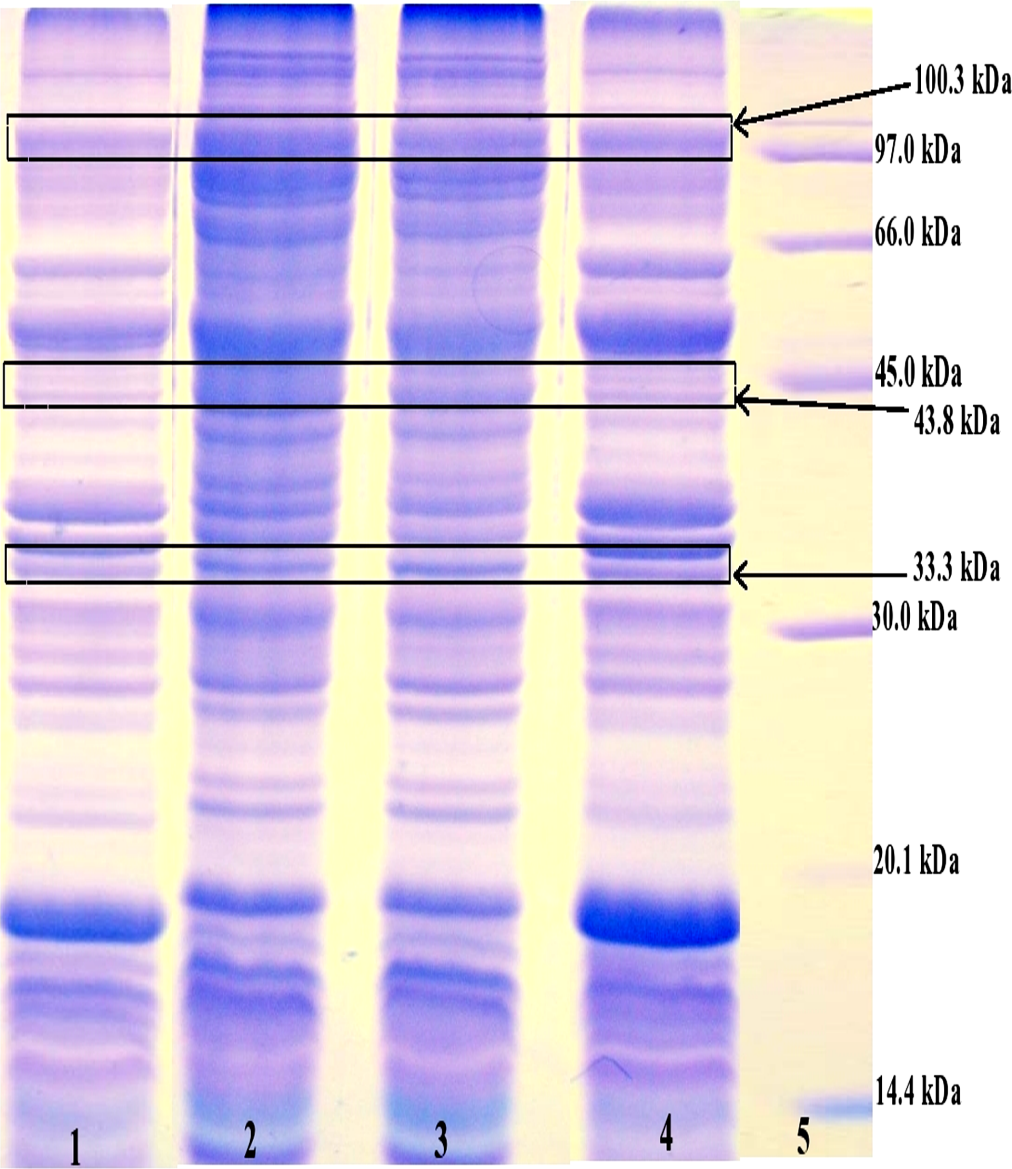
Expression of the specific protein bands (33.3 kDa, 43.8 kDa and 100.3 kDa) in the OMPs of *S*. *sonnei* strains. Lane 1: OMPs of *S*. *sonnei* ATCC 25931, Lane 2: OMPs of clinical isolate SH039, Lane 3: OMPs of clinical isolate SH040, Lane 4: OMPs of clinical isolate SH080, Lane 5: Low molecular weight marker.

The study demonstrated that three specific protein bands (33.3, 43.8 and 100.3 kDa) were found to be not cross-reacting with tested non-shigellosis sera against both isotypes of antibodies (IgA and IgG). These specific and antigenic bands (33.3, 43.8, 100.3 kDa) were also present in other clinical isolate of *S*. *sonnei* and in reference strain ATCC 25931 as shown in Fig 3.

## Discussion

Immunoglobulins of different classes are important effectors of specific humoral immunity. A previous study reported that acquired immunity to *Shigella* produced by the secretory IgA and serum immunoglobulins, which are specific to some major bacterial pathogenic factors[19]. Another study also reported that the IgM antibody titers against OMPs were not significantly increased in patients infected with *Shigella* [19, 20]. As such, this study was conducted to focus on both IgA and IgG isotypes.

In this study, the immunoblot profile of OMPs of *S*. *sonnei* was studied to determine the host humoral immune response in shigellosis patients against the antigens in the OMPs. The analysis of this study showed variation in the host immune response between the sera tested. For example, the sera from patients SS001 and SS002 gave the highest number of antigenic bands against anti-human IgA, in which a total of 11 antigenic bands were recognized. Whereas, with serum SS003 and SS004, seven and four antigenic bands were observed respectively. These individual variations in antibody response against the antigen in the OMPs seen in this study showed that host humoral immune system responded differentially in each infection. Studies have proven that, genetics factors play a major role to determine the susceptibility of a species to an infection with enteric pathogen [21, 22].

A study reported that high level of IgA antibody was frequently detected between 10 and 20 days after the onset of *Shigella* infection [23]. Studies have also demonstrated that the total IgA in serum increases between 2 to 4 days after the onset of the disease and reached its highest level within the first week [24–26]. In our study, the recognition of antigens with IgA antibodies in sera will be useful in diagnosing acute cases. An earlier study also reported the serum IgG antibody responses to proteins of *Shigella* spp. detected by immunoblot [27]. This experiment revealed that some antigenic proteins were exclusively recognized by IgA and IgG. In OMPs of *S*. *sonnei* few protein bands were exclusively recognized by anti-human IgA (37.8 kDa and 63.2 kDa) or anti-human IgG (38.5 kDa and 46.3 kDa). These findings suggest that the detection of antibody of a single immunoglobulin isotype may not be diagnostically sufficient. The combination of biomarkers in developing diagnostic test will be useful in decreasing false negative result for accurate diagnosis of shigellosis.

Several studies have been conducted previously on the discovery of biomarkers in *Shigella* spp. A study conducted using major outer membrane proteins (MOMPs) showed that the protein band sized ranging from 35 to 38 kDa and few other MOMPs act as major antigens in the induction of immune response in shigellosis [28, 29]. Similarly, in our study, serum immune response against the proteins of 33.0, 35.0 and 37.8 kDa in size was detected in the tested sera.

The immunogenic profile detected with *Shigella* sera were compared to the profile obtained with sera from other enteric infections. This is to determine the specific antigenic protein(s) expressed by *S*. *sonnei* by eliminating the cross-reactive proteins. In this study, we found that the three protein bands (33.3, 43.8 and 100.3 kDa) were recognized by shigellosis sera did not cross reacted with sera from other enteric infections. These protein candidates have potential to be used as a diagnostic biomarker for early diagnosis of shigellosis since the sera were collected from the early stage of *Shigella* infected patients. This study also showed that these protein bands were also present in the clinical isolates and reference strain of *S*. *sonnei* studied when SDS-PAGE was run. The occurrence of these bands in all the clinical isolate is also an indicator that these proteins are important for the virulence of *S*. *sonnei* during infection. In summary, this study has demonstrated the presence of antigenic protein bands of *S*. *sonnei*, which may be potential candidates for further evaluation towards the development of rapid diagnostic test. Further studies with a wider range of serum samples are needed to ensure the specificity and reliability of these antigenic bands as diagnostic biomarkers.

## Supporting information

S1 Table Dot-EIA result of OMPs (clinical isolate SH080) probed with patient’s sera of *S*. *sonnei* and other related infection. (PDF)
