# Supplementary material for: Outer membrane proteins analysis of Shigella sonnei and evaluation of their antigenicity in Shigella infected individuals
Source: PLoS One. 2017 Aug 28;12(8):e0182878. doi: 10.1371/journal.pone.0182878 (PMC5573271; doi:10.1371/journal.pone.0182878)
Supplement: S1 Table — (PDF) [file pone.0182878.s001.PDF]

Serum stock number	Dot-EIA with *S. sonnei* OMPs	
	IgA	IgG
SS001<br>*Shigella sonnei* 1	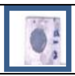	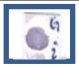
SS002<br>*Shigella sonnei* 2	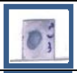	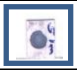
SS003<br>*Shigella sonnei* 3	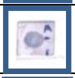	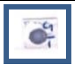
SS004<br>*Shigella sonnei* 4	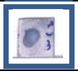	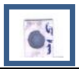
SASP01<br>*Salmonella* spp	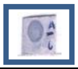	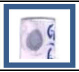
E01<br>EPEC	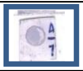	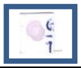
ST01<br>*Salmonella* Typhi	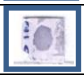	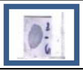
AH01<br>*Aeromonas hydrophila*	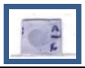	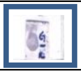
CAMPY01<br>*Campylobacter jejuni*	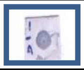	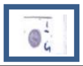
Normal sera	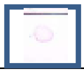	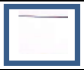
